# The Revolution in Medicine

**Published:** 1887-02

**Authors:** 


### BOOK NOTICES AND REVIEWS.

The Revolution in Medicine. By John H. Clark, M. D. London: Keene & Ashwell. 1886.

This little duodecimo is the seventh Hahnemannian Oration, delivered October 5th, 1886, at the London Homœopathic Hospital, where the author is physician and lecturer on Materia Medica. It contains the freshest presentation of the old but always interesting story of the rise of Homœopathy, and of the persecutions of its immortal founder, Samuel Hahnemann.

